# Human Immunodeficiency Virus Type 1 Nef Inhibits Autophagy through Transcription Factor EB Sequestration

**DOI:** 10.1371/journal.ppat.1005018

**Published:** 2015-06-26

**Authors:** Grant R. Campbell, Pratima Rawat, Rachel S. Bruckman, Stephen A. Spector

**Affiliations:** 1 Division of Infectious Diseases, Department of Pediatrics, University of California San Diego, La Jolla, California, United States of America; 2 Rady Children’s Hospital, San Diego, California, United States of America; Vanderbilt University School of Medicine, UNITED STATES

## Abstract

HIV Nef acts as an anti-autophagic maturation factor through interaction with beclin-1 (BECN1). We report that exposure of macrophages to infectious or non-infectious purified HIV induces toll-like receptor 8 (TLR8) and BECN1 dependent dephosphorylation and nuclear translocation of TFEB and that this correlates with an increase in autophagy markers. RNA interference for *ATG13*, *TFEB*, *TLR8*, or *BECN1* inhibits this HIV-induced autophagy. However, once HIV establishes a productive infection, TFEB phosphorylation and cytoplasmic sequestration are increased resulting in decreased autophagy markers. Moreover, by 7 d post-infection, autophagy levels are similar to mock infected controls. Conversely, although Nef deleted HIV similarly induces TFEB dephosphorylation and nuclear localization, and increases autophagy, these levels remain elevated during continued productive infection. Thus, the interaction between HIV and TLR8 serves as a signal for autophagy induction that is dependent upon the dephosphorylation and nuclear translocation of TFEB. During permissive infection, Nef binds BECN1 resulting in mammalian target of rapamycin (MTOR) activation, TFEB phosphorylation and cytosolic sequestration, and the inhibition of autophagy. To our knowledge, this is the first report of a virus modulating TFEB localization and helps to explain how HIV modulates autophagy to promote its own replication and cell survival.

## Author Summary

Under basal conditions, the mammalian target of rapamycin (MTOR) phosphorylates transcription factor EB (TFEB) resulting in its cytoplasmic retention. When MTOR is inhibited, TFEB is dephosphorylated and translocated to the nucleus where it increases autophagy and lysosomal gene expression. As human immunodeficiency virus type 1 (HIV) Nef acts as an anti-autophagic maturation factor through interaction with beclin-1 (BECN1), we investigated the role of Nef and TFEB in the modulation of autophagy during HIV infection of human macrophages. We found that upon exposure to HIV, macrophages elicited an autophagic response through a toll-like receptor 8 (TLR8) and BECN1 dependent dephosphorylation and nuclear translocation of TFEB. However, once HIV infection is established, phosphorylation and cytoplasmic sequestration of TFEB as well as autophagy revert to pre-infection levels. Moreover, this reversion is dependent upon the presence of HIV Nef. Collectively, the data suggests that the interaction between HIV and TLR8 serves as a signal for autophagy induction that is dependent upon the dephosphorylation and nuclear translocation of TFEB. Once HIV establishes a productive infection, Nef binds BECN1 resulting in MTOR activation, TFEB phosphorylation and cytosolic sequestration and the inhibition of autophagy.

## Introduction

As an obligate intracellular parasite, human immunodeficiency virus type 1 (HIV) survival is dependent upon its ability to exploit host cell machinery for replication and dissemination, and to evade intrinsic cellular processes and defenses that may limit viral replication and pathogenesis including macroautophagy (hereafter referred to as autophagy) [1]. Autophagy is a degradation pathway whereby cytosolic double membrane-bound compartments termed autophagosomes engulf and sequester cytoplasmic constituents such as sub-cellular organelles and microbial pathogens. These autophagosomes then fuse with lysosomes (organelles that contain an array of hydrolytic enzymes capable of degrading almost any biomolecule) forming autophagolysosomes (autolysosomes), resulting in the degradation of the engulfed components. Evidence of integrated and co-regulated roles of lysosomes and autophagosomes has emerged from the discovery of an overarching lysosomal regulatory gene network (CLEAR, Coordinated Lysosomal Expression and Regulation) and its master regulator, the basic helix-loop-helix leucine zipper transcription factor EB (TFEB). During starvation, cells activate a transcriptional program coordinated by TFEB that controls all major steps of the autophagic pathway, including autophagosome formation, autophagosome-lysosome fusion, and substrate degradation [2]. In resting cells, mammalian target of rapamycin (MTOR) complex 1 (MTORC1) is active and phosphorylates Ser^142^ and Ser^211^ of TFEB that results in retention of the transcription factor in the cytoplasm through binding of 14-3-3 proteins that occlude a nuclear localization sequence thereby promoting the cytoplasmic sequestration of TFEB [3–5]. When MTORC1 is inhibited or inactivated, the balance shifts towards dephosphorylation of Ser^142^ and Ser^211^ resulting in diminished interactions between TFEB and 14-3-3 proteins that reveals the nuclear localization sequence leading to nuclear accumulation of TFEB and the expression of autophagosomal and lysosomal proteins [3, 5]. Thus, TFEB is a regulator of autophagic clearance and is at the crossroads of the regulatory mechanisms that coordinate both the autophagy and lysosomal pathways.

Of the more than 35 human autophagy-associated genes currently known to be involved in autophagy, ten are now known to be essential for HIV replication [reviewed in 1]. However, although HIV may require the early stages of autophagy, it must control the antiviral proteolytic and degradative late stages of autophagy to avoid its degradation. The current data suggest that HIV has developed mechanisms to inhibit autophagic degradation involving the HIV negative regulatory factor (Nef) [6]. In HIV-infected macrophages, Nef inhibits the proteolytic stages of autophagy by binding to amino acids 267–284 in the beclin-1 (BECN1) evolutionarily conserved domain [7]. This is the same region that is necessary and sufficient for BECN1 to bind glioma-associated oncogene pathogenesis-related 2 (GLIPR2) [7], a protein that associates with lipid rafts at the cytosolic leaflet of the Golgi membrane [8] and that negatively regulates autophagy by sequestering BECN1 to the Golgi complex [7]. Despite the down-modulation of autophagy by HIV, inducers of autophagy including 1α,25-dihydroxycholecalciferol [9, 10], amino acid starvation [11], hydroxamate histone deacetylase inhibitors [12], sirolimus [7, 9], toll-like receptor (TLR) 8 ligands [13], romidepsin [12], and a cell-permeable autophagy-inducing peptide termed Tat–beclin (derived from the region of BECN1 that interacts with HIV Nef and conjugated to the basic region of HIV Tat) [7], overcome the imposed phagosome maturation block leading to inhibition of viral replication.

As HIV Nef acts as an anti-autophagic maturation factor through interaction with BECN1, we investigated the role of Nef and TFEB in the modulation of autophagy during HIV infection of macrophages. The present data suggest that the interaction between HIV and TLR8 serves as a signal for autophagy induction that is dependent upon the dephosphorylation and nuclear translocation of TFEB. Once HIV establishes a productive infection, Nef inhibits autophagy by binding BECN1 resulting in TFEB phosphorylation and cytosolic sequestration. These findings, to our knowledge, are the first that report a virus modulating TFEB localization and help to explain how HIV modulates autophagy to promote its own replication and cell survival.

## Results

### HIV induces autophagy in human primary macrophages

**Fig 1:**
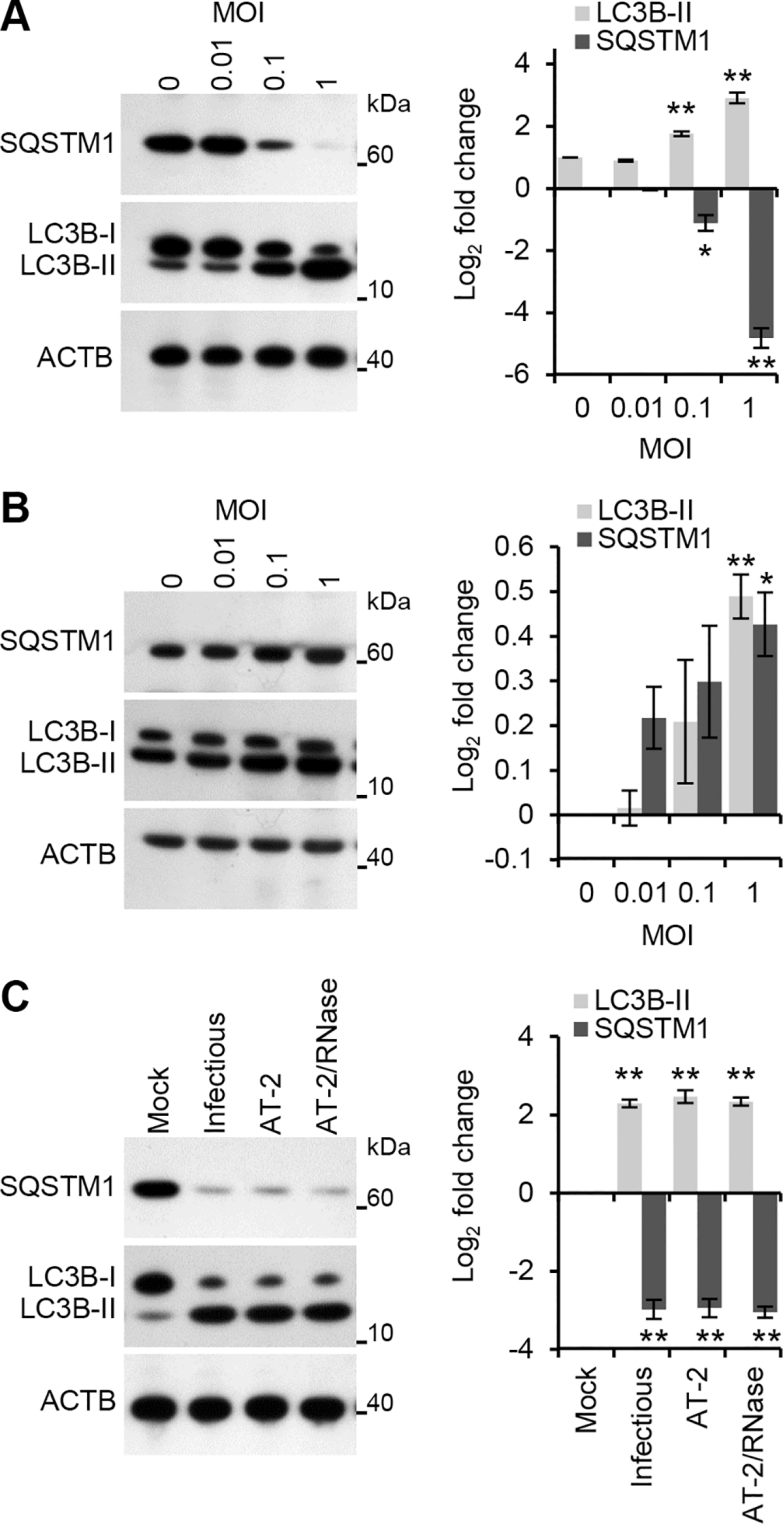
HIV induces autophagy in human macrophages. (A) Macrophages were exposed to increasing concentrations of cell-free RNase/DNase I treated iodixanol velocity gradient purified HIV for 24 h, harvested, lysed and analyzed for endogenous LC3B, SQSTM1, and ACTB by Western blotting. *Left*, a representative blot is shown. *Right*, densitometric analysis of immunoblots from independent donors presented as means ± s.e.m., *n* = 3. (B) Macrophages were exposed to increasing concentrations of cell-free RNase/DNase I treated iodixanol velocity gradient purified HIV for 24 h in the presence of pepstatin A, harvested, lysed and analyzed for endogenous LC3B, SQSTM1, and ACTB by Western blotting. *Left*, a representative blot is shown. *Right*, densitometric analysis of immunoblots from independent donors presented as means ± s.e.m., *n* = 3. (C) Macrophages were exposed to mock, infectious, AT-2-inactivated, or RNase/DNase I treated AT-2-inactivated iodixanol velocity gradient purified HIV_Ba-L_ for 24 h. Cells were then harvested, lysed and analyzed for endogenous LC3B and SQSTM1 by Western blotting. *Left*, a representative blot is shown. *Right*, densitometric analysis of immunoblots from independent donors presented as means ± s.e.m., *n* = 3.

Whereas exposure of primary human macrophages to HIV envelope proteins has no autophagy inducing effect, exposure to MOLT-4 cells chronically infected with HIV induces autophagy by day 3 post-co-culture [14]. However, the autophagic status of macrophages after exposure to purified HIV virions is unknown. Therefore, the effect of HIV on autophagy induction in human macrophages was determined using RNase/DNase I treated virus purified through an iodixanol velocity gradient (purified HIV), which effectively separates extracellular proteins and microvesicles from the virus (S1 Fig) [15]. During autophagy, cytosolic microtubule-associated protein 1 light chain 3 beta (LC3B)-I is converted to LC3B-II by a ubiquitin-like system that involves autophagy related (ATG) 7, ATG3 and the ATG12–ATG5 complex. The ATG12–ATG5 complex ligates LC3B-II to the nascent autophagosome membrane through phosphatidylethanolamine with the LC3B-II associated with the inner membrane degraded after fusion of the autophagosome with lysosomes. Therefore, the conversion of LC3B-I to LC3B-II and its turnover is an indicator of autophagy induction and flux [16]. Exposure of macrophages to purified HIV led to a significant dose-dependent increase in LC3B-II after 24 h (Fig 1A) in the absence of significant cytotoxic effects (*P* > 0.05; S2A Fig).

To verify that the increase in LC3 lipidation represented increased autophagic flux rather than an accumulation of LC3B-II, the degradation of the polyubiquitin-binding protein sequestosome 1 (SQSTM1) was also quantified. Inhibition of autophagy leads to an increase in SQSTM1 protein levels while autolysosomes degrade SQSTM1- and LC3-positive bodies during autophagic flux [17]. Purified HIV induced a significant dose-dependent decrease in SQSTM1 protein levels corresponding to the stimulation of autophagic flux at 24 h post-infection (Fig 1A). To confirm that the decreased SQSTM1 levels result from enhanced degradation via autophagy and not through diminished transcription, autophagosome degradation was inhibited with the lysosomal protease inhibitor pepstatin A. Both SQSTM1 and LC3B-II were significantly increased in the presence of pepstatin A indicative of autophagic flux (Fig 1B) in the absence of significant cytotoxic effects (*P* > 0.05; S2B Fig).

**Fig 2:**
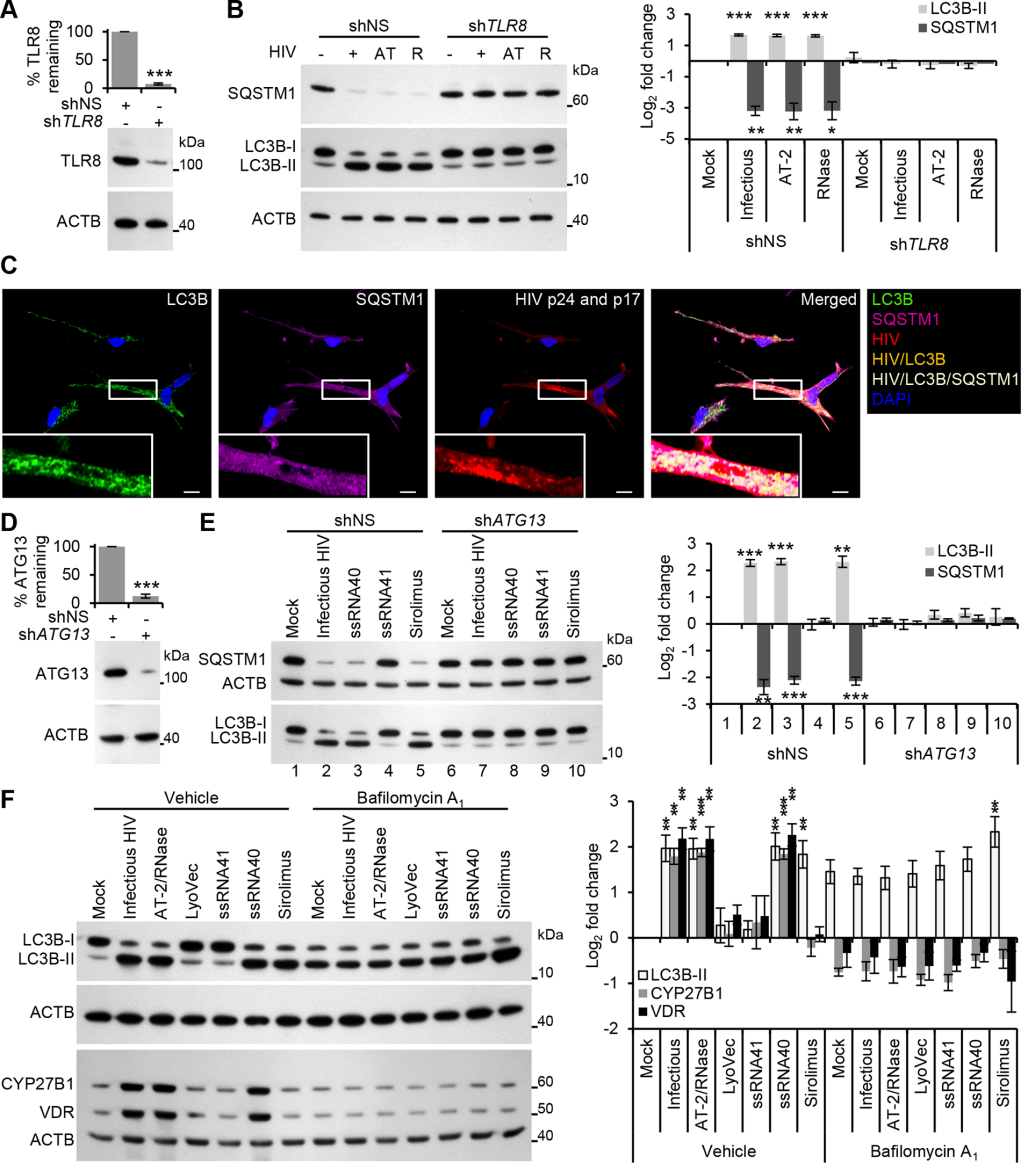
HIV induces autophagy in human macrophages through TLR8. (A) Macrophages were transduced with non-specific scrambled shRNA (shNS), or *TLR8* shRNA (sh*TLR8*) and analyzed for TLR8 expression. *Bottom*, a representative blot is shown. *Top*, densitometric analysis of immunoblots from independent donors presented as means ± s.e.m., *n* = 4. (B) Macrophages transduced with shNS or sh*TLR8* from (A) were exposed to infectious HIV (+), AT-2-inactivated HIV (AT), or RNase/DNase I treated AT-2-inactivated HIV (R) or mock infected (-) for 24 h, harvested, lysed and analyzed for endogenous LC3B and SQSTM1 by Western blotting. *Left*, a representative blot is shown. *Right*, densitometric analysis of immunoblots from independent donors presented as means ± s.e.m., *n* = 4. (C) Representative fluorescence microscopy images of HIV-infected macrophages which were fixed, permeabilized then stained with 4',6-diamidino-2-phenylindole (DAPI; *blue*) and antibody to LC3B (*green*), SQSTM1 (*magenta*), HIV p24 and p17 (*red*). Co-localization of HIV and LC3B is represented by *orange* pixels, and co-localization of HIV, SQSTM1, and LC3B is represented by *cream* pixels. Scale bars indicate 10 *μ*m. (D) Macrophages were transduced with shNS or *ATG13* shRNA (sh*ATG13*) and analyzed for ATG13 expression. *Bottom*, a representative blot is shown. *Top*, densitometric analysis of immunoblots from independent donors presented as means ± s.e.m., *n* = 4. (E) Macrophages transduced with shNS or sh*ATG13* from (D) were exposed to infectious HIV, 5 *μ*g/mL ssRNA40, 5 *μ*g/mL ssRNA41, or 100 nmol/L sirolimus for 24 h, harvested, lysed and analyzed for endogenous LC3B and SQSTM1 by Western blotting. *Left*, a representative blot is shown. *Right*, densitometric analysis of immunoblots from independent donors presented as means ± s.e.m., *n* = 4. (F) Macrophages were pretreated with 100 nmol/L bafilomycin A_1_ then exposed to mock, infectious, or RNase/DNase I treated AT-2-inactivated purified HIV, LyoVec, 5 *μ*g/mL ssRNA41, 5 *μ*g/mL ssRNA40, or 100 nmol/L sirolimus for 24 h, harvested, lysed and analyzed for endogenous LC3B, CYP27B1 and VDR by Western blotting. *Left*, a representative blot is shown. *Right*, densitometric analysis of immunoblots from independent donors presented as means ± s.e.m., *n* = 3.

In order to infect productively a target cell, HIV envelope protein gp120 binds to CD4, triggering conformational changes in gp120 that ultimately leads to the fusion of the viral and target cell membranes allowing entry of the viral capsid. Within uninfected CD4^+^ T cells, the fusogenic activity of gp41 induces autophagy [18] leading to the induction of apoptosis [19]. In contrast, uninfected or infected macrophages do not undergo Env-mediated autophagy or apoptosis [14]. In addition to this route of entry, HIV can also enter macrophages through CD4-independent macropinocytosis [20] or a macropinocytosis-like mechanism (the pathway of HIV endocytic entry in macrophages [PHEEM]) [21]. Following entry through a CD4-independent pathway, the uridine rich HIV long terminal repeat (LTR) single-stranded RNA, which contains multiple pathogen-associated molecular patterns (PAMPs), can be recognized by the pattern recognition receptor (PRR) TLR8 expressed in macrophage endosomes [22, 23]. Previously, we demonstrated that ssRNA40, a GU-rich ssRNA derived from the HIV LTR, induces autophagy in human macrophages through a TLR8-dependent mechanism involving vitamin D, and the expression of both the vitamin D (1,25D3) receptor, and cytochrome P450, family 27, subfamily B, polypeptide 1 (CYP27B1), which 1α-hydroxylates the inactive form of vitamin D3, 25-hydroxycholecalciferol (25D3), into the biologically active metabolite 1,25D3, and (VDR) [13]. Therefore, we investigated whether productive infection was required for the induction of autophagy using 2,2′-dithiodipyridine (AT-2)-treated HIV. AT-2 inactivates the infectivity of retroviruses by covalently modifying the nucleocapsid zinc finger motifs (S3 Fig). Exposure of macrophages to AT-2-inactivated HIV for 24 h led to a significant increase in LC3B-II (*P* = 0.0045) and significant degradation of SQSTM1 (*P* = 0.006). Interestingly, there was no significant difference in either LC3B lipidation or SQSTM1 between AT-2-inactivated HIV and exposure to infectious HIV indicating that productive infection is not required for the HIV-mediated induction of autophagy (*P* > 0.05; Fig 1C). To determine the role of TLR8, RNA interference (RNAi) of *TLR8* was employed. *TLR8* silencing (Fig 2A) significantly inhibited HIV-mediated LC3B lipidation and degradation of SQSTM1 (Fig 2B) in the absence of significant cytotoxic effects (*P* > 0.05; S2D Fig) suggesting that TLR8 is the mediator of HIV-induced autophagy in macrophages. Although we observed an increase in LC3B lipidation and an increase in SQSTM1 degradation, ingestion of pathogens through TLR1/2, TLR2/6, and TLR4 can trigger the recruitment of LC3B-II to single-membrane phagosomes in a process termed LC3-associated phagocytosis (LAP) [24]. As opposed to canonical autophagy, LAP is the receptor-mediated internalization of extracellular cargo that occurs without the formation of a double membrane. The receptor triggers the ligation of LC3 to the phagosome. These LC3-positive SQSTM1-negative phagosomes then fuse with lysosomes and rapidly mature into a phagolysosomes. The pre-initiation complex that is required for autophagy is dispensable for LAP, as LC3B-II deposition at the phagosome proceeds normally in the absence of RB1-inducible coiled-coil 1, ATG13 and unc-51 like autophagy activating kinase 1 (ULK1) proteins [25]. The ability of HIV and TLR8 ligands to initiate LAP is unknown. Therefore, we analyzed whether HIV exposed macrophages contain more SQSTM1 and LC3B dual positive autophagosomes or LC3B-positive SQSTM1-negative phagosomes harboring HIV particles using confocal immunofluorescence microscopy (Fig 2C). We observed SQSTM1 and LC3B dual positive puncta that were also positive for HIV (cream pixels), but failed to observe the presence of LC3B-positive SQSTM1-negative puncta harboring HIV (orange pixels). We then investigated whether HIV or ssRNA40 induces TLR8-mediated LAP using RNAi for *ATG13*. Silencing of *ATG13* (Fig 2D) abrogated LC3B lipidation and SQSTM1 degradation following ssRNA40 (*P* < 0.0005) and HIV (*P* < 0.005) exposure for 24 h suggesting that the autophagy pre-initiation complex is required for LC3B lipidation in response to TLR8 triggering (Fig 2E).

TLR8 is both phylogenetically and structurally similar to TLR7, activates similar signaling pathways, and is located within endosomes. The signaling pathway of TLR7 is dependent upon endosomal acidification (maturation) [26, 27]. Bafilomycin A_1_ is an inhibitor of the vacuolar H^+^ ATPase, effectively inhibiting endosomal acidification (maturation) and thus the signaling pathways of endosomal TLR3, TLR7 and TLR9 [26–28]. Importantly, although bafilomycin A_1_ blocks the fusion of autophagosomes with lysosomes, leading to an accumulation of autophagosomal structures it has no direct effect on the conversion of LC3B-I to LC3B-II. For instance, sirolimus, an inhibitor of MTOR that initiates autophagy independently of endosome acidification, induces significant LC3B-II accumulation in the presence of bafilomycin A_1_ (*P* = 0.015; Fig 2F) indicative of induced autophagy but arrested flux [29], in the absence of significant cytotoxic effects (*P* > 0.05; S2F Fig). However, pretreatment of macrophages with bafilomycin A_1_ resulted in the inhibition of HIV and ssRNA40 induced LC3B lipidation (*P* > 0.2; Fig 2F). Moreover, bafilomycin A_1_ also inhibited the increase in TLR8 mediated CYP27B1 (*P* > 0.28) and VDR (*P* > 0.37) expression, downstream effectors of TLR8 signaling that are required for TLR8 mediated autophagy induction [13]. Collectively, these results demonstrate that the induction of autophagy in macrophages by HIV does not require productive infection, and is mediated through a TLR8 signaling pathway that requires endosomal maturation.

**Fig 3:**
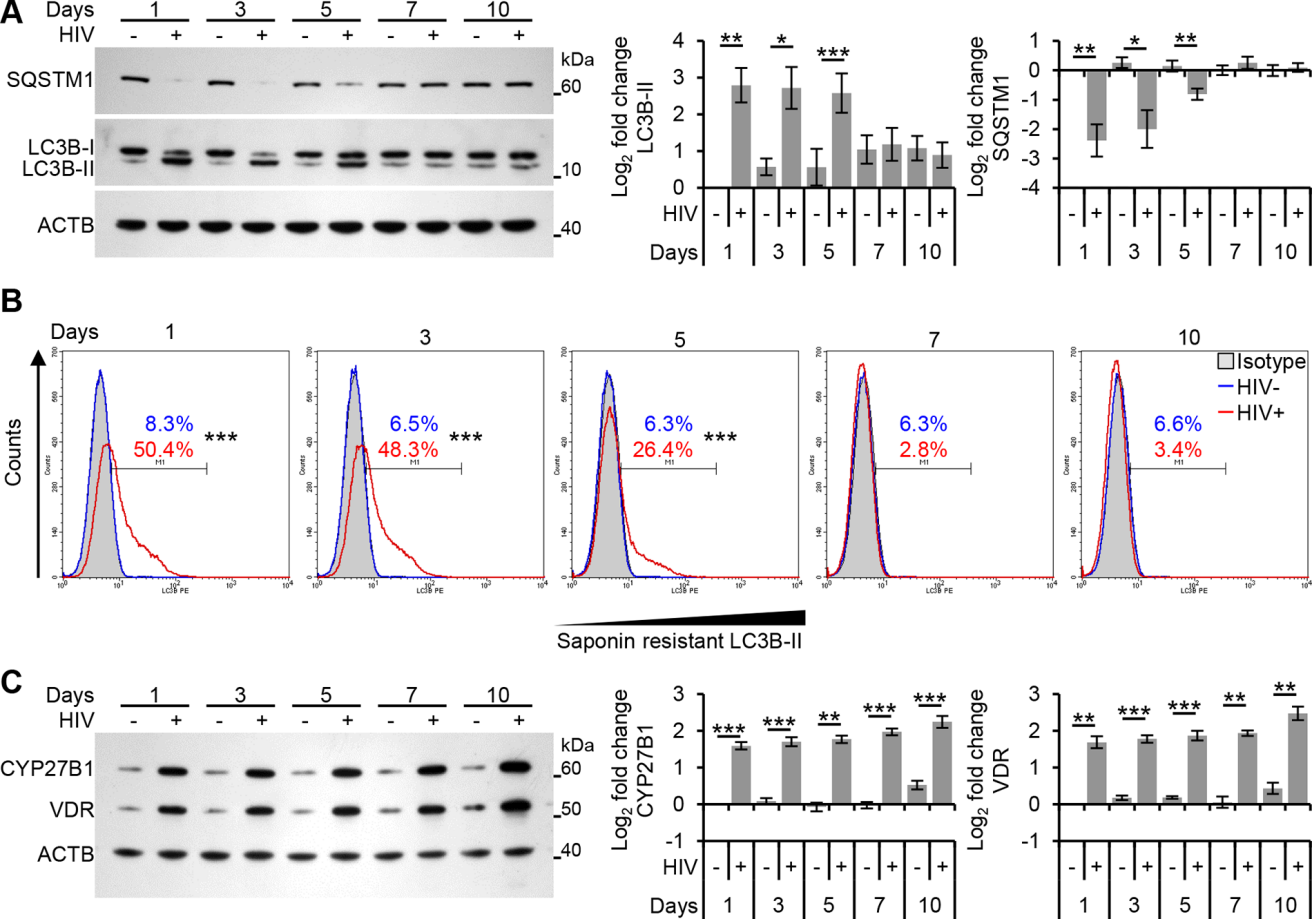
Autophagy is decreased at later time points post-infection in human macrophages. Macrophages were infected with HIV and cells harvested at 1, 3, 5, 7, and 10 days post-infection. (A) Cells were analyzed for endogenous LC3B and SQSTM1 by Western blotting. *Left*, a representative blot is shown. *Right*, densitometric analysis of immunoblots from independent donors presented as means ± s.e.m., *n* = 6. (B) Cells were harvested then subjected to flow cytometry analysis of saponin-resistant LC3B-II in macrophages. Representative histograms of cells displaying saponin-resistant LC3B-II are shown. (C) Cells were harvested, lysed then analyzed for CYP27B1 and VDR by Western blotting. *Left*, a representative blot is shown. *Right*, densitometric analysis of immunoblots from independent donors presented as means ± s.e.m., *n* = 6.

We next assessed the autophagic status of primary macrophages after long-term infection with replication competent virus. There was a significant increase in LC3B lipidation at both 24 h and 72 h post-infection (*P* < 0.05; Fig 3A). By 5 d post-infection, although still significantly increased, LC3B lipidation had appreciably decreased and by 7 d was the same as the mock-infected controls (*P* > 0.05). Similarly, HIV infection induced a significant decrease in SQSTM1 protein levels (*P* < 0.001) corresponding to the stimulation of autophagic flux at 24 h post-infection. SQSTM1 protein levels became progressively greater from 3 d to 5 d post-infection and by 7 d post-infection were the same as the mock-infected controls (*P* > 0.05; Fig 3A). When autophagosomes are formed, LC3B redistributes from a soluble diffuse cytosolic pattern to an insoluble autophagosome-associated vacuolar pattern [30, 31] allowing the quantification of autophagosome-associated LC3B-II in human macrophages using saponin resistance and flow cytometry [30]. Staining for endogenous LC3B in saponin washed macrophages revealed that the percentage of cells containing a saponin resistant fraction was significantly increased at both 24 and 72 h post-infection (*P* < 0.001; Fig 3B). By 5 d post-infection, the number of cells expressing saponin resistant fractions had decreased, but was still significant (*P* = 0.0002) and by 7 d was the same as the mock-infected controls. Despite the downregulation of autophagy markers observed by 7 d post-infection, CYP27B1 and VDR were still significantly upregulated indicating that TLR8 is still sufficiently engaged at these late time posts post-infection (Fig 3C) in the absence of significant cytotoxic effects (*P* > 0.05; S2G Fig).

### TFEB mediates HIV-induced autophagy

**Fig 4:**
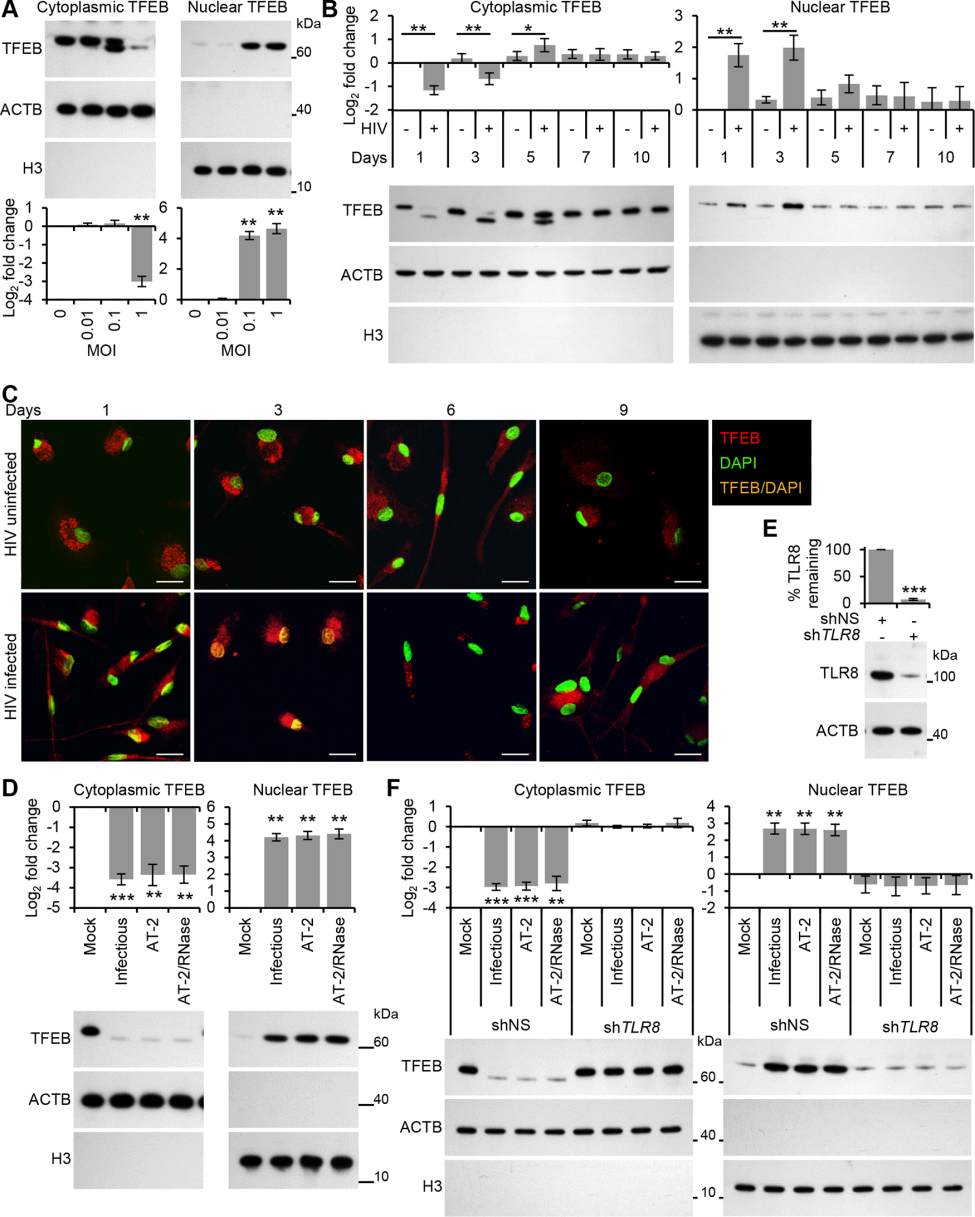
HIV induces TFEB nuclear localization in human macrophages through TLR8. (A) Macrophages were exposed to increasing concentrations of purified HIV for 24 h, harvested, lysed, fractionated for cytoplasmic and nuclear content, and analyzed for TFEB, ACTB and H3 histone by Western blotting. *Top*, a representative blot is shown. *Bottom*, densitometric analysis of immunoblots from independent donors presented as means ± s.e.m., *n* = 3. (B) Macrophages were infected with HIV and at 1, 3, 5, 7, and 10 days post-infection cells were harvested, lysed and fractionated for cytoplasmic and nuclear content, and analyzed for TFEB, ACTB and H3 histone by Western blotting. *Bottom*, a representative blot is shown. *Top*, densitometric analysis of immunoblots from independent donors presented as means ± s.e.m., *n* = 6. (C) Representative fluorescence microscopy images of HIV-infected macrophages which were fixed, permeabilized then stained with 4',6-diamidino-2-phenylindole (DAPI; *green*) and antibody to TFEB (*red*) at the indicated times post-infection. Co-localization of TFEB and DAPI staining is represented in the image by *orange* pixels. Scale bars indicate 20 *μ*m. (D) Macrophages were exposed to mock, infectious, AT-2-inactivated, or RNase/DNase I treated AT-2-inactivated iodixanol velocity gradient purified HIV_Ba-L_ for 24 h, harvested, lysed fractionated for cytoplasmic and nuclear content, and analyzed for TFEB, ACTB and H3 histone by Western blotting. *Bottom*, a representative blot is shown. *Top*, densitometric analysis of immunoblots from independent donors presented as means ± s.e.m., *n* = 3. (E) Macrophages were transduced with non-specific scrambled shRNA (shNS), or *TLR8* shRNA (sh*TLR8*) and analyzed for TLR8 expression. *Bottom*, a representative blot is shown. *Top*, densitometric analysis of immunoblots from independent donors presented as means ± s.e.m., *n* = 4. (F) Macrophages transduced with shNS or sh*TLR8* from (E) were exposed to mock, infectious, AT-2-inactivated, or RNase/DNase I treated AT-2-inactivated purified HIV for 24 h. Cells were then harvested, lysed, fractionated for cytoplasmic and nuclear content, and analyzed for TFEB, ACTB and H3 histone by Western blotting. *Top*, a representative blot is shown. *Bottom*, densitometric analysis of immunoblots from independent donors presented as means ± s.e.m., *n* = 4.

To investigate the role of TFEB in regulating autophagy activation upon exposure to HIV, we initially examined TFEB localization using immunoblotting (Fig 4A). The results show that TFEB localizes predominantly in the cytoplasm of uninfected macrophages. Moreover, almost all TFEB ran at a higher molecular size than in the HIV-exposed samples suggesting that a substantial fraction of TFEB is phosphorylated under basal conditions. Exposure to purified HIV for 24 h led to a significant and dose-dependent dephosphorylation and activation of TFEB as monitored by its more rapid mobility in sodium dodecyl sulfate polyacrylamide gel electrophoresis, and increased nuclear accumulation (Fig 4A). We then assessed the effect of long-term productive HIV infection on TFEB localization. The dephosphorylation and activation of TFEB lasted until at least 72 h post-infection and by 5 d post-infection, although levels were elevated, TFEB was localized predominantly to the cytoplasmic fraction. By 7 d TFEB localization was similar to the mock-infected controls (Fig 4B). To confirm further the nuclear translocation of TFEB, we monitored TFEB sub-cellular localization using confocal immunofluorescence microscopy. Macrophages were cultured in the presence of HIV and the sub-cellular distribution of TFEB was evaluated using 4',6-diamidino-2-phenylindole nuclear staining and an anti-TFEB antibody (Fig 4C). In untreated macrophages, TFEB localized predominantly to the cytoplasm whereas in HIV exposed macrophages TFEB translocated to the nucleus by 72 h. However, by 6 d post-infection, this effect had dissipated and TFEB localization was again similar to the mock-infected controls (Fig 4C).

**Fig 5:**
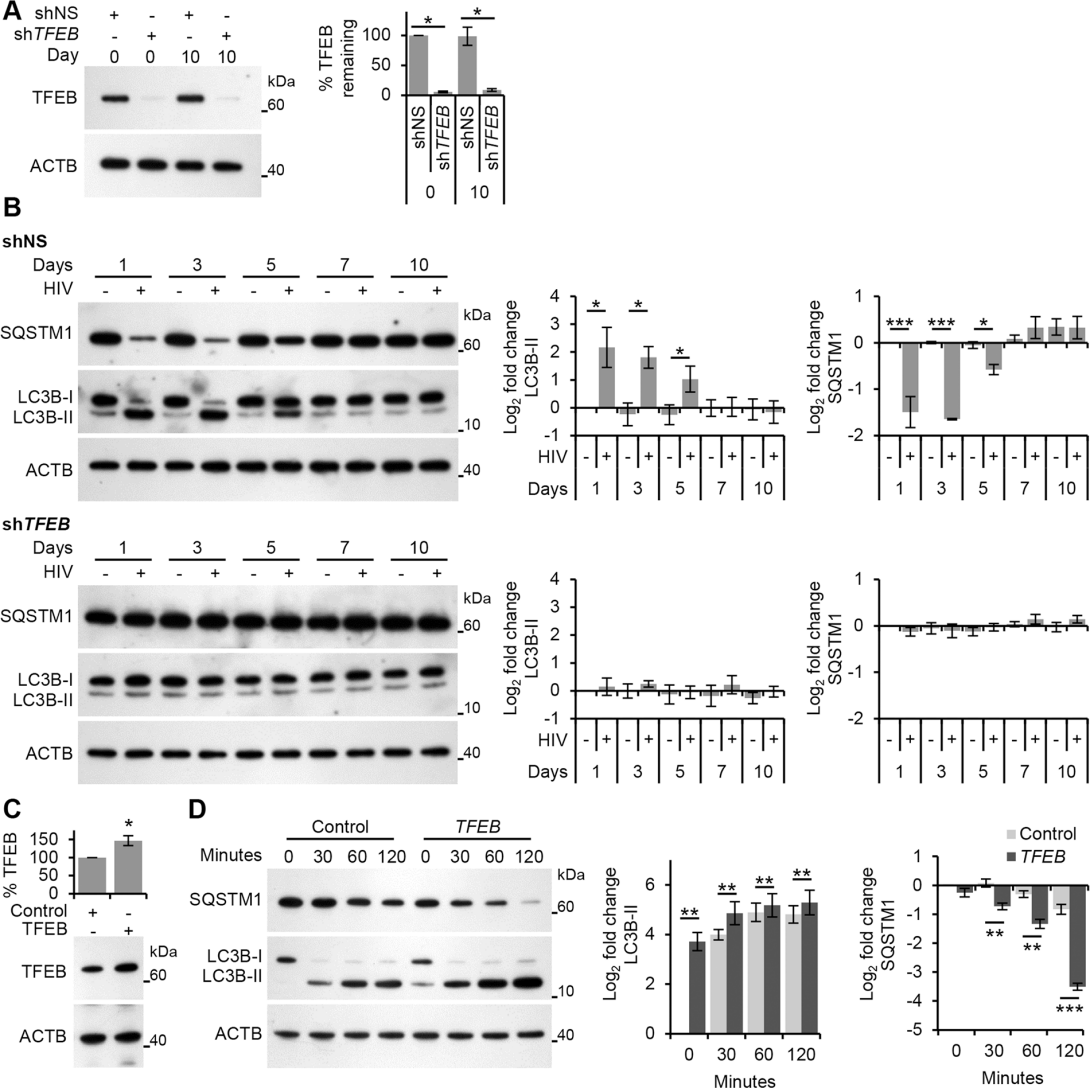
HIV-mediated autophagy induction is dependent upon TFEB. (A) Macrophages transduced with non-specific scrambled shRNA (shNS), or *TFEB* shRNA (sh*TFEB*) were infected with HIV for 10 days. Cells were lysed and analyzed for TFEB and ACTB content by Western blotting. *Left*, a representative blot is shown. *Right*, densitometric analysis of immunoblots from independent donors presented as means ± s.e.m., *n* = 4. (B) At 1, 3, 5, 7, and 10 days post-infection cells from (A) were harvested, lysed, and analyzed for endogenous LC3B and SQSTM1 by Western blotting. *Left*, representative blots are shown. *Right*, densitometric analysis of immunoblots from independent donors presented as means ± s.e.m., *n* = 4. (C) Macrophages transduced with non-specific cDNA (Control), or *TFEB* cDNA (*TFEB*) then exposed to HIV. Cells were lysed and analyzed for TFEB and ACTB content by Western blotting. *Bottom*, a representative blot is shown. *Top*, densitometric analysis of immunoblots from independent donors presented as means ± s.e.m., *n* = 4. (D) Transduced cells from (C) were exposed to purified HIV for the indicated time (minutes) harvested, lysed, and analyzed for endogenous LC3B and SQSTM1 by Western blotting. *Left*, representative blots are shown. *Right*, densitometric analysis of immunoblots from independent donors presented as means ± s.e.m., *n* = 4.

As permissive HIV infection is not required for the induction of autophagy (Fig 1C), we investigated whether productive infection was required for the dephosphorylation and nuclear translocation of TFEB at the early time points. Treatment with AT-2-inactivated purified HIV led to the dephosphorylation and nuclear localization of TFEB by 24 h indicating that productive infection was not necessary (Fig 4D). Furthermore, silencing of *TLR8* (Fig 4E) abrogated the HIV-mediated dephosphorylation and nuclear translocation of TFEB (Fig 4F). To assess the role of TFEB in HIV-mediated autophagy, macrophages were transduced with short hairpin RNA (shRNA) specific to *TFEB*, followed by exposure to HIV. *TFEB* silencing (Fig 5A) significantly reduced both the lipidation of LC3B and the degradation of SQSTM1 in macrophages post-HIV infection (Fig 5B) in the absence of significant cytotoxic effects (*P* > 0.05; S2I Fig). We then tested whether TFEB overexpression also regulated autophagy in macrophages using a lentivirus overexpressing TFEB. Transient TFEB overexpression (Fig 5C) significantly increased LC3B lipidation and SQSTM1 degradation post HIV exposure indicating that TFEB enhances HIV induced autophagic flux in macrophages (Fig 5D).

**Fig 6:**
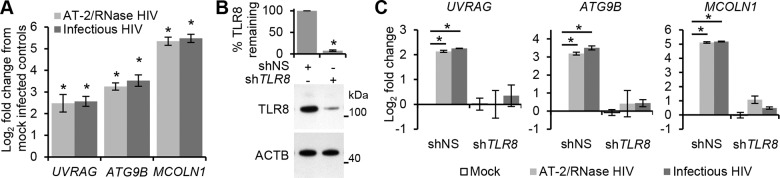
HIV-mediated autophagy and lysosomal gene expression is dependent upon TFEB. (A) qRT-PCR analysis of mRNA expression of autophagy (*UVRAG* and *ATG9B*) and lysosomal (*MCOLN1*) genes 24 h post-exposure to mock, infectious, or RNase/DNase I treated AT-2-inactivated purified HIV. Data are reported as mean ± s.e.m., *n* = 4. (B) Macrophages were transduced with non-specific scrambled shRNA (shNS), or *TLR8* shRNA (sh*TLR8*) and analyzed for TLR8 expression. *Bottom*, a representative blot is shown. *Top*, densitometric analysis of immunoblots from independent donors presented as means ± s.e.m., *n* = 4. (C) Macrophages from (B) were exposed to mock, infectious, or RNase/DNase I treated AT-2-inactivated purified HIV for 24 h. Cells were harvested and qRT-PCR for *UVRAG*, *ATG9B*, *MCOLN1* was performed. Data were normalized to the shNS mock infected control for each gene. Bar charts are reported as mean ± s.e.m., *n* = 4.

Finally, we analyzed the transcriptional activity of TFEB post-HIV exposure. For this, we exposed macrophages to both infectious HIV and AT-2-inactivated HIV and assessed the expression of the known TFEB targets *ATG9B*, *UV radiation resistance associated gene* (*UVRAG*) (both autophagy genes), and *mucolipin 1* (*MCOLN1*) (a lysosomal gene) after 24 h using qRT-PCR. Both infectious HIV and AT-2-inactivated HIV increased the transcription of *ATG9B*, *UVRAG*, and *MCOLN1* suggesting activation of TFEB by HIV does not require productive infection (Fig 6A). Moreover, when *TLR8* was silenced, expression of these genes post-HIV exposure was similar to the mock-infected controls (Fig 6C). Collectively, these data suggest that exposure of macrophages to HIV induces TLR8-dependent TFEB dephosphorylation and nuclear translocation that induces autophagy, and that this is not dependent upon a productive infection.

### HIV-induced TFEB translocation is BECN1 dependent

**Fig 7:**
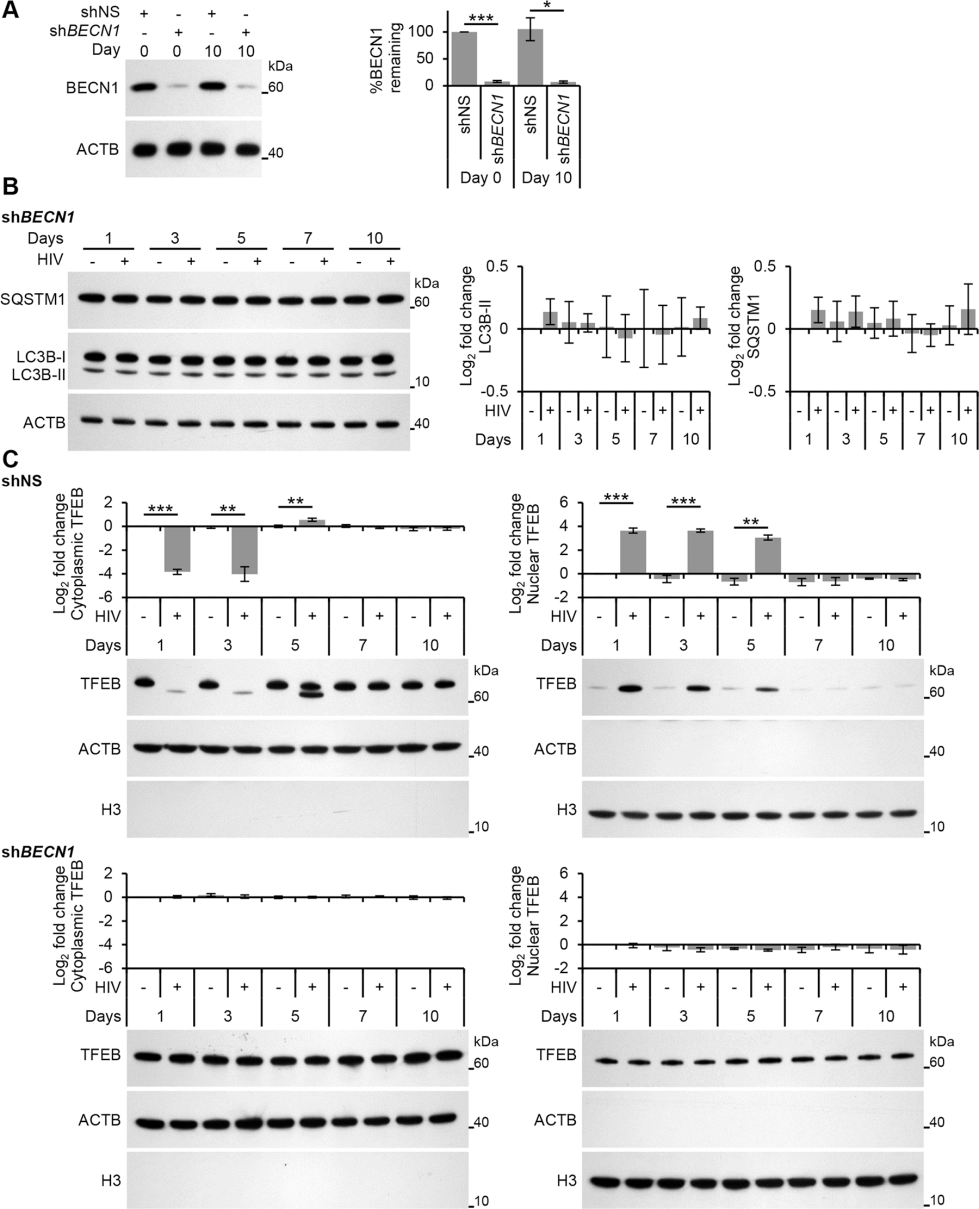
HIV-mediated autophagy induction and nuclear translocation of TFEB is dependent upon BECN1. (A) Macrophages transduced with non-specific scrambled shRNA (shNS), or *BECN1* shRNA (sh*BECN1*) were infected with HIV for 10 days. Cells were lysed and analyzed for BECN1 and ACTB content by Western blotting. *Left*, a representative blot is shown. *Right*, densitometric analysis of immunoblots from independent donors presented as means ± s.e.m., *n* = 4. (B) At 1, 3, 5, 7, and 10 days post-infection cells from (A) were harvested, lysed and analyzed for endogenous LC3B, SQSTM1, and ACTB by Western blotting. *Left*, a representative blot is shown. *Right*, densitometric analysis of immunoblots from independent donors presented as means ± s.e.m., *n* = 4. (C) At 1, 3, 5, 7, and 10 days post-infection cells from (A) were harvested, lysed, fractionated for cytoplasmic and nuclear content, and analyzed for TFEB, ACTB and H3 histone by Western blotting. *Left*, a representative blot is shown. *Right*, densitometric analysis of immunoblots from independent donors presented as means ± s.e.m., *n* = 4.

Autophagy is well integrated into the innate immune system with PAMP induced PRR signaling activating autophagy [32]. For example, TLR4 signaling leads to ubiquitination of BECN1 by E3 ubiquitin protein ligase tumor necrosis factor receptor-associated factor 6 (TRAF6) which releases it from its inhibitor, B cell lymphoma 2 (BCL2) [33]. In the context of HIV, TLR8 signaling by the HIV LTR RNA stimulates enhanced binding of BECN1 to phosphoinositide-3-kinase (PIK3C3) forming the PIK3C3 kinase complex, which is essential for the induction of autophagosome formation at the vesicle elongation step [13]. In both cases, BECN1 is essential for the induction of autophagy. Therefore, we investigated whether the dephosphorylation and nuclear translocation of TFEB post-HIV infection was dependent upon BECN1. Macrophages transduced with shRNA specific to *BECN1* were exposed to HIV. As expected, *BECN1* silencing significantly inhibited autophagic flux as measured by significant reductions in both the lipidation of LC3B and the degradation of SQSTM1 in macrophages post-HIV infection (Fig 7B). *BECN1* silencing also abrogated TFEB dephosphorylation and nuclear localization at all time points (Fig 7C).

### HIV Nef inhibits TFEB nuclear translocation and autophagic flux

**Fig 8:**
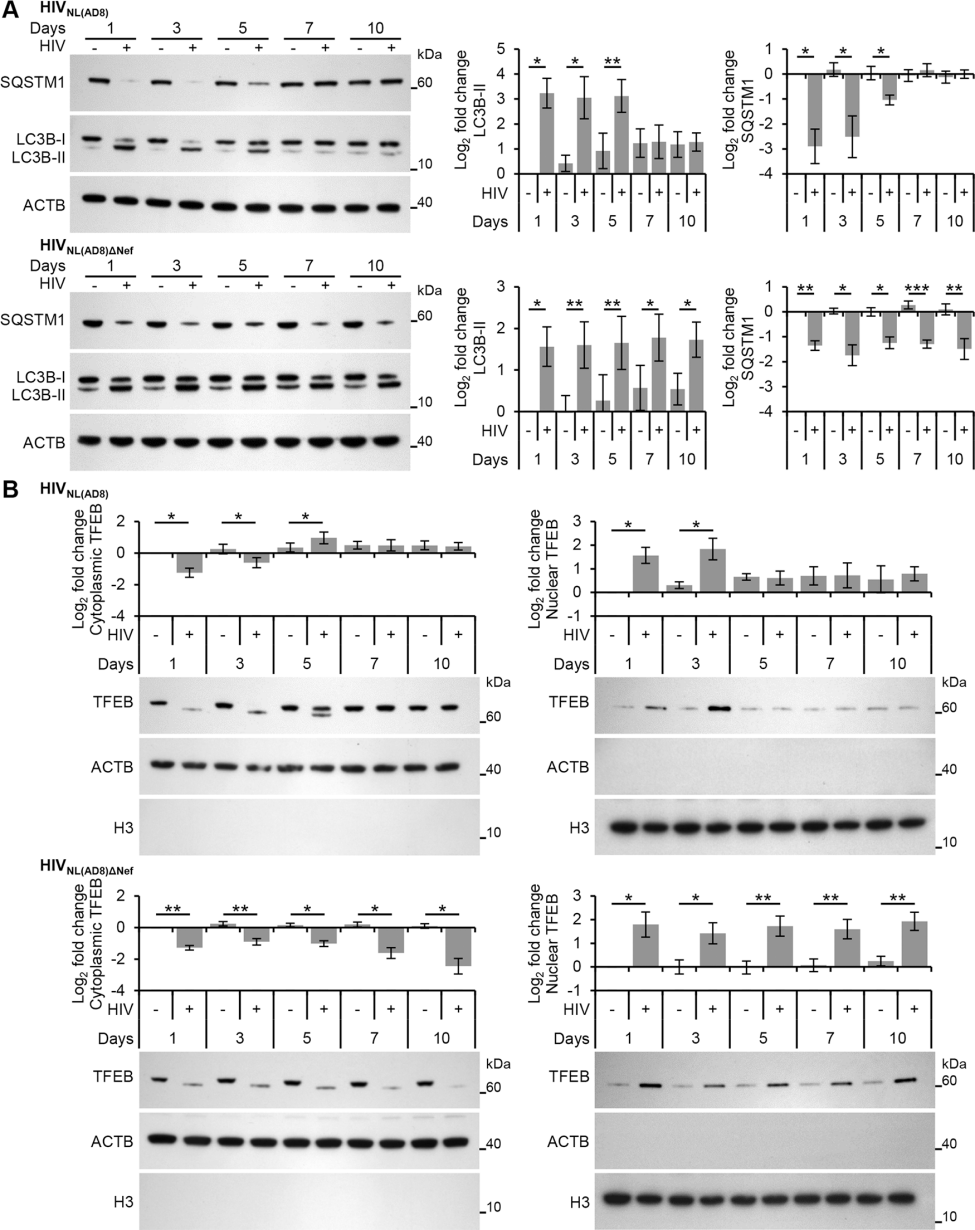
HIV-mediated autophagy induction and nuclear translocation of TFEB is inhibited by HIV Nef. (A) Macrophages were infected with HIV_NL(AD8)_ or HIV_NL(AD8)ΔNef_. At 1, 3, 5, 7, and 10 days post-infection cells were harvested, lysed and analyzed for endogenous LC3B, SQSTM1, and ACTB by Western blotting. *Left*, representative blots are shown. *Right*, densitometric analysis of immunoblots from independent donors presented as means ± s.e.m., *n* = 4. (B) Macrophages were infected with HIV_NL(AD8)_ or HIV_NL(AD8)ΔNef_. At 1, 3, 5, 7, and 10 days post-infection cells were harvested, lysed, fractionated for cytoplasmic and nuclear content, and analyzed for TFEB, ACTB and H3 histone by Western blotting. *Left*, representative blots are shown. *Right*, densitometric analysis of immunoblots from independent donors presented as means ± s.e.m., *n* = 4.

HIV, in addition to using basal autophagy for its own replication [1], utilizes the Nef protein to protect itself against autophagic degradation [6]. Nef acts as an anti-autophagic maturation factor through interaction with BECN1 [6], and is required for efficient viral replication and HIV pathogenicity. Therefore, we investigated whether Nef was responsible for the down regulation of autophagy observed during permissive HIV infection. Both complete HIV (HIV_NL(AD8)_) and Nef deleted HIV (HIV_NL(AD8)ΔNef_), significantly increased LC3B lipidation and SQSTM1 degradation at 24 h and 72 h post-HIV-exposure (Fig 8A). As was the case for HIV_Ba-L_, the levels of LC3B-II and SQSTM1 in HIV_NL(AD8)_ treated cells were similar to that found in the mock infected controls by 5 d post-exposure and this trend continued through 10 d post-exposure. In contrast, macrophages infected with the Nef deleted HIV_NL(AD8)ΔNef_ maintained significantly greater LC3B lipidation and SQSTM1 degradation at all time points (Fig 8A). Importantly, both HIV_NL(AD8)_ and HIV_NL(AD8)ΔNef_ demonstrated extracellular p24 release over the 10 d infection protocol indicating replication competent virus (S4A Fig). These data suggest that once HIV establishes a productive infection, it inhibits autophagy through a Nef-dependent mechanism. Based on our observations that TLR8 agonists inhibit HIV replication through the induction of autophagy, and that silencing *TLR8* inhibits HIV-mediated autophagy, we sought to determine whether the inhibition of HIV-induced autophagy would rescue viral replication of Nef deficient HIV at later time points. Silencing of *TLR8* resulted in a marked increase in the release of HIV p24 antigen at later time points by both HIV_NL(AD8)_ and HIV_NL(AD8)ΔNef_ (*P* < 0.05; S4B Fig).

As the dephosphorylation, activation and nuclear translocation of TFEB was required for the induction of autophagy post-HIV exposure, we examined the effect of HIV_NL(AD8)ΔNef_ on TFEB localization using immunoblotting. Unlike exposure to wild-type HIV, which resulted in the transient dephosphorylation and nuclear translocation of TFEB, infection of macrophages with HIV_NL(AD8)ΔNef_ resulted in the dephosphorylation and increased nuclear translocation of TFEB by 24 h post-infection that lasted throughout the experiment until cells were terminated at 10 d post-infection (Fig 8B). These findings indicate that Nef is required to inhibit the TLR8-mediated induction of autophagy that occurs through subsequent rounds of HIV infection at later time points. This is of key significance as the Nef-dependent actions of inhibiting autophagosome formation and preventing their maturation to autolysosomes [6] may spare the virus from early degradation.

## Discussion

The interaction between viruses and autophagy can be bi-directional and may function to both degrade viruses and/or promote viral replication. The known antiviral effects of autophagy include virophagy (the degradation of cytoplasmic viral constituents), the activation of innate and adaptive immunity (through the delivery of viral antigens to endosomal TLRs or major histocompatibility complex class I and II, respectively), and promotion of cell survival. The positive-strand RNA viruses dengue virus, poliovirus, and hepatitis C virus are good examples of viruses for which autophagy promotes and facilitates viral replication, all of which utilize the maturation of autophagosomes for their replication [34]. In the present study, we investigated the mechanisms by which HIV infection influences autophagy during primary infection of human primary macrophages. It was previously demonstrated that the fusogenic function of HIV gp41 was sufficient to trigger autophagy in CD4^+^ T cells, but not in macrophages [14, 18], and that ssRNA40 derived from the HIV LTR induces autophagy in macrophages through TLR8 in the absence of any other viral antigen [13, 32]. In the present study, we identify that HIV induces autophagy in macrophages through a mechanism that is dependent upon endosomal acidification, TLR8, ATG13, BECN1, and the dephosphorylation and nuclear translocation of TFEB. Moreover, we demonstrate that HIV induces autophagy in macrophages independently of viral replication. HIV is not alone in this respect. Vesicular stomatitis Indiana virus protein G induces virophagy independently of viral replication through interactions with TLR7 on *Drosophilia* cells [35], and nonvirulent measles virus hemagglutinin induces autophagy through the golgi-associated PDZ and coiled-coil motif containing protein (GOPC) and BECN1 through CD46 on the surface of HeLa cells [36]. TLR signaling reduces the binding of BECN1 to BCL2 via the TLR adaptor proteins myeloid differentiation primary response 88 (MYD88) and toll-like receptor adaptor molecule 1 (TICAM1) by recruiting BECN1 into the TLR-signaling complex leading to the inhibition of MTOR and the induction of autophagy [37]. Importantly, exposure of macrophages to HIV resulted in TFEB-dependent autophagy activation in the absence of apoptosis or cell death, suggesting that macrophages respond to HIV by activating virophagy.

During HIV infection, the expression of TLR8 within peripheral blood monocytes decreases with disease progression. Moreover, monocytes from HIV-infected individuals produce less tumor necrosis factor following TLR8 activation than those from uninfected individuals while successfully inhibiting HIV infection [38]. These monocyte responses are negatively correlated with CD4^+^ T cell numbers and positively associated with HIV viral load [39]. The ability of cells to respond strongly to a TLR8 agonist in the presence of high HIV viremia suggests that ongoing chronic immune activation may be continuously driven by HIV-encoded PAMPs. However, tolerance is not induced towards TLR8 agonists [39, 40]. Persistent immune activation during HIV infection contributes to the pathogenesis of disease by disturbing the functional organization of the immune system with induction of high levels of cytokines and chemokines. Therefore, chronic stimulation of the innate immune system by TLR ligands may result in the chronic production of proinflammatory cytokines which drive disease progression through generalized immune activation [41]. Indeed, HIV induces both pro-interleukin-1 beta (IL1B) expression and its subsequent cleavage into bioactive IL1B through NLRP3 inflammasome activation in monocytes and macrophages in an infection-independent process that requires clathrin-mediated endocytosis and recognition of the viral ssRNA by TLR8 [42, 43]. Supporting this model is the association of a single-nucleotide polymorphism in *TLR8* (*TLR8* A1G; rs3764880) which confers a significant protective effect against HIV disease progression [44].

Cytoplasmic TFEB is located both in the cytosol and on the lysosomal surface, where it interacts with MTORC1 and the lysosomal nutrient sensing (LYNUS) machinery [4]. Our findings are consistent with the model in which inhibition, rather than activation, of MTORC1 induces TFEB nuclear translocation. Indeed, pharmacological inhibition of MTOR using Torin 1, chloroquine, Sa1A, and transfection with mutant Rag proteins all result in the nuclear accumulation of TFEB [4]. Interestingly, other members of the basic-helix–loop–helix family of transcription factors, such as microphthalmia-associated transcription factor (MITF) and transcription factor binding to immunoglobulin heavy constant mu enhancer 3 (TFE3), the sequences of which are closely related to TFEB, seem to be regulated by a similar mechanism [3]. It will be interesting to investigate whether HIV or TLR signaling affects these proteins.

We found that in the absence of Nef the HIV-induced TFEB nuclear translocation and induction of autophagy was present at 10 d post-infection. We also found that Nef-deficient HIV replicated less efficiently. It is likely that Nef-deficient HIV replicates less efficiently due to its inability to overcome autophagic degradation. Nef plays a major role in the inhibition of the proteolytic stages of autophagy in macrophages by binding and sequestering BECN1 through its ^174^DD motif [6]. Although this motif is required for CD4 downregulation and interactions with the V_1_ domain of the vacuolar H^+^-ATPase [45], it is unlikely that it influences H^+^-ATPase assembly or activity thereby inhibiting autophagosome acidification or autophago-lysosome fusion as endosome acidification is independent of Nef [46]. However, as Nef binds the evolutionarily conserved domain of BECN1 (the same region that allows GLIPR2 to bind and sequester BECN1, thereby suppressing autophagy [7]), it is possible that Nef negatively regulates autophagy by sequestering BECN1. Autophagy initiation is thought to be strictly dependent upon phosphatidylinositol 3-phosphate (PtdIns(3)P) synthesis by PIK3C3, in complex with BECN1 at the trans-Golgi network. However, whereas all cellular BECN1 is associated with PIK3C3, 50% of PIK3C3 is not associated with BECN1, and is localized to endosomes [47]. Moreover, PtdIns(3)P has been implicated in the regulation of autophagy upstream of MTOR via PIK3C3 [48]. Therefore, by sequestrating BECN1, Nef may promote the localization of free PIK3C3 to endosomes, and in our amino acid rich environment, activate MTOR [48]. This phosphorylates TFEB and inhibits autophagy while simultaneously stimulating mRNA translation through the phosphorylation of RPS6KB1 and EIF4E1B. Although additional evidence is needed to support this model, consistent with this hypothesis, BECN1 silencing also inhibited the dephosphorylation and nuclear translocation of TFEB upon HIV exposure, indicating that MTOR had not been inactivated. HIV is not alone in its ability to suppress autophagy. As viruses have evolved under pressure from autophagic degradation within their eukaryotic hosts, it is not surprising that many have evolved strategies to circumvent autophagy, and a striking number of them target BECN1. Human cytomegalovirus TRS1 protein [49], African swine fever virus A179L protein [50], herpes simplex virus type 1 ICP34.5 protein [51], human herpesvirus 8 orf16 protein [52], and murid herpesvirus 68 M11 protein [53] all bind BECN1 and block autophagosome biogenesis. In contrast to the DNA viruses that inhibit autophagosome generation, RNA viruses seem to stabilize autophagosomes by preventing their degradation. For instance, the influenza A virus M2 protein binds BECN1 and inhibits autophagosome maturation [54]. The current data suggest that HIV, through Nef, falls into both categories, inhibiting both autophagosome biogenesis and maturation.

The potent inhibitory effects of autophagy on HIV replication [1, 7, 9, 12, 13] combined with the ability of HIV to inhibit autophagy serve to illustrate its importance in the cellular antiviral response [1, 6, 11]. To our knowledge, this is the first report demonstrating viral regulation of the autophagy master regulator TFEB. Understanding how HIV and other viruses such as influenza and herpes viruses inhibit autophagy may lead to the development of broad-spectrum antiviral drugs that restore autophagy through pharmacological means during viral infection with the aim of eliminating the virus. In the case of HIV, this is both attractive and novel as autophagy works at the host cellular level to improve intracellular killing of both replicating and non-replicating HIV while resistance is unlikely to develop. Dissecting the molecular mechanisms by which HIV utilizes autophagy has the potential to lead to the identification of novel drug candidates to treat HIV infection.

## Materials and Methods

### Ethics statement

Venous blood was drawn from HIV seronegative subjects using a protocol that was reviewed and approved by the Human Research Protections Program of the University of California, San Diego (Project 09–0660) in accordance with the requirements of the Code of Federal Regulations on the Protection of Human Subjects (45 CFR 46 and 21 CFR 50 and 56). Written informed consent was obtained from all blood donors prior to their participation.

### Cell cultures and cell transduction

Monocyte derived macrophages were generated from whole blood of HIV seronegative donors as previously described [13]. All experiments were performed in RPMI 1640 supplemented with 10% (v/v) charcoal/dextran treated, heat-inactivated fetal bovine serum (FBS; Gemini Bio-Products), 10 ng/mL macrophage colony stimulating factor (Peprotech), and 40 ng/mL 25-hydroxycholecalciferol (Sigma) (growth media). Sirolimus and bafilomycin A_1_ were obtained from Sigma and LC Laboratories respectively. LyoVec, ssRNA40, and ssRNA41 were obtained from Invivogen. Cell death was estimated using the lactate dehydrogenase (LDH) Cytotoxicity Detection Kit^PLUS^ (Roche).

Lentiviral transduction of macrophages with MISSION pLKO.1-puro lentiviral vectors containing shRNAs targeting *ATG13* (SHCLNV-NM_ 014741/TRCN0000172507), *BECN1* (SHCLNV-NM_003766/TRCN0000033551 and TRCN0000299864), *TFEB* (SHCLNV-NM_007162/TRCN0000013108), *TLR8* (SHCLNV-NM_138636/TRCN0000359320), or scrambled non-target negative control (SHC002V) was performed according to the manufacturer's protocol (Sigma). Macrophages were transduced with non-specific scrambled shRNA (shNS) or target shRNA and selected for using puromycin (Gibco). Five days later, cells were analyzed for target gene silencing and used in experiments.

### Virus preparation

HIV_Ba-L_ (HIV) was obtained through the NIH AIDS Research and Reference Reagent Program from Dr. Suzanne Gartner and Dr. Robert Gallo [55, 56] and was expanded and concentrated as previously described [57]. Virus was then subjected to a 6 to 18% iodixanol velocity gradient in 1.2% increments using OptiPrep (60% [w/v] iodixanol; Sigma) diluted in DPBS essentially as previously described [15]. Briefly, supernatants were laid over the gradient and centrifuged for 1.5 h at 37,500 rpm (250,000 × g at *r*_max_) in an SW41 Ti rotor using an L8-70M ultracentrifuge (both Beckman Coulter). Fourteen gradient fractions were collected and analyzed for both total protein and HIV p24 content by SDS-PAGE and immunoblot analysis. HIV titers were determined on phytohemagglutinin-P-stimulated peripheral blood mononuclear cells (PBMC) as described previously using the Alliance HIV p24 antigen ELISA (Perkin Elmer) [57] and multiplicity of infection confirmed using TZM-bl cells obtained through the NIH AIDS Research and Reference Reagent Program, from Drs John C. Kappes and Xiaoyun Wu and Tranzyme Inc. [58]. R5-tropic, replication-competent HIV-1 strain HIV_NL(AD8)_ and its derivative Nef deleted mutant HIV_NL(AD8)ΔNef_ were generated by transient transfection of HEK293T cells separately with pNL(AD8) [59] or pNL(AD8)ΔNef [60] (both kind gifts from Olivier Schwartz, Pasteur Institute, France) using the calcium phosphate method [61]. Virus was harvested at 48 and 72 h post-transfection, filtered through a 0.22 *μ*m polyethersulfone filter (Millipore), and purified as described above. For inactivation with 100 *μ*mol/L AT-2 (Sigma), HIV was treated for 1 h at 37°C. For RNase treatment virus stock was resuspended in 1 mL 10 mmol/L 2-amino-2-(hydroxymethyl)-1,3-propanediol hydrochloride (1:1; pH 7.4), 100 mmol/L NaCl and treated with 80 U RQ1 DNase I (Promega) and 10 U RNase I (Ambion) for 1 h at 37°C. At the conclusion of AT-2 and/or RNase/DNase I treatments, agents were removed by ultrafiltration at 4°C using a Vivaspin 2 with a 300 kDa cutoff (Sartorius Stedim) followed by purification as described above. Control virus preparations were sham treated and processed in parallel with inactivated samples. Mock infection preparations were prepared from uninfected IL2 treated PBMC supernatants and processed in parallel with HIV stocks. For all procedures, frozen virus stocks were quickly thawed at 37°C in a water bath and cells exposed to 90 *μ*L of a 10^6.2^ TCID_50_/mL HIV per 10^5^ cells for 3 h, washed then incubated in growth media for the times indicated.

### Immunoblotting

The following antibodies were used: *β*-actin (ACTB; AC-74), TLR8 (4C6), VDR (N-terminal) (all Sigma), BECN1 (#3738), Histone H3 (H3; D1H2) (both Cell Signaling), LC3B (NB100-2220; Novus Biologicals), TFEB (Bethyl Laboratories), SQSTM1 (ab56416; Abcam), CYP27B1 (H-90; Santa Cruz Biotechnology), and HIV p24 (P131; Abcam). Whole cell lysates were prepared using 20 mmol/L 4-(2-hydroxyethyl)-1-piperazineethanesulfonic acid, 1 mmol/L ethylenediaminetetraacetic acid (both Gibco), 150 mmol/L NaCl, 1% (v/v) 4-(1,1,3,3-tetramethylbutyl)phenyl-polyethylene glycol (both Sigma) and 1% (v/v) Halt protease and phosphatase inhibitor cocktail (Thermo Scientific). The NE-PER nuclear and cytoplasmic extraction reagent kit supplemented with 1% (v/v) Halt protease and phosphatase inhibitor cocktail (both Thermo Scientific) was used for cell lysis and extraction of separate cytoplasmic and nuclear protein fractions. For immunoblot analyses, cell lysates were resolved using 2-[bis(2-hydroxyethyl)amino]-2-(hydroxymethyl)propane-1,3-diol buffered 12% polyacrylamide gel (Novex) and transferred to low fluorescence 0.2 *μ*m pore-size polyvinylidene difluoride membranes (Thermo Scientific), followed by detection with alkaline phosphatase tagged secondary antibodies (Invitrogen) and 0.25 mmol/L disodium 2-chloro-5-(4-methoxyspiro[1,2-dioxetane-3,2′-(5-chlorotricyclo[3.3.1.1^3.7^]decan])-4-yl]-1-phenyl phosphate supplemented with 5% (v/v) Nitro-Block II (both Applied Biosystems). Relative densities of the target bands compared to the reference bands (ACTB for total lysates and cytoplasmic fractions and H3 for nuclear fractions) were analyzed using ImageJ (NIH). Each sample was normalized to the vehicle then log_2_ transformed.

### qRT-PCR

mRNA quantification was measured by qRT-PCR using the LightCycler 1.5 Instrument and the FastStart RNA Master SYBR Green I kit (both Roche Applied Science). PCR reactions were carried out in a 20 *μ*L mixture composed of 3.25 mmol/L Mn(CH_3_COO)_2_, 0.5 *μ*mol/L of each primer, 1 *μ*L sample and 1-fold LightCycler RNA Master SYBR Green I. Primers were synthesized by Integrated DNA Technologies: *MCOLN1* sense 5’- AGGGGCTCTGGGCTACC-3’, antisense 5’- GCCCGCCGCTGTCACTG-3’; *ATG9B* sense 5’-TGTGCTCACCGTCTACGAC-3’, antisense 5’-GGGAGGTAGTGCATGTGGG-3’; *UVRAG* sense 5’-ATGCCAGACCGTCTTGATACA-3’, antisense 5’-TGACCCAAGTATTTCAGCCCA-3’; *polymerase (RNA) II (DNA directed) polypeptide A* (*POLR2A*) sense 5’-GCACCACGTCCAATGACAT-3’, antisense 5’-GTGCGGCTGCTTCCATAA-3’. Reaction parameters were as follows: 61°C at 20 min followed by 95°C at 30 s followed by 45 cycles of 10 s, 95°C; 10 s, 60°C; 15 s, 72°C. Data were analyzed using the Pfaffl method [62]. The ratio between the target gene mRNA and *POLR2A* (the reference gene) was then calculated and normalized so that mRNA expression in mock infected cells equals 1.00. Data were then log_2_ transformed.

### Flow cytometry

Intracellular staining and analysis of endogenous saponin resistant LC3B was performed as previously described [9, 10, 30] using rabbit anti-LC3B (D11; Cell Signaling) followed by phycoerythrin (PE) conjugated goat anti-rabbit IgG (Santa Cruz Biotechnology).

### Microscopy

The following primary antibodies were used: TFEB (ab2636), SQSTM1 (ab31545) HIV-1 p24 (ab155836), HIV-1 p55/p17 (ab2581; all Abcam), LC3B (2775; Cell Signaling). The following secondary antibodies were used: Alexa Fluor 647-conjugated donkey anti-goat, Alexa Fluor 647-conjugated donkey anti-mouse, Alexa Fluor 568-conjugated donkey anti-sheep, Alexa Fluor 488-conjugated donkey anti-rabbit (all Molecular Probes). Cells were fixed in Dulbecco's phosphate-buffered saline supplemented with 4% (w/v) paraformaldehyde for 10 min, permeabilized with 0.2% (v/v) 4-(1,1,3,3-tetramethylbutyl)phenyl-polyethylene glycol for 10 min, probed with primary antibodies for 30 min, washed, then probed with secondary antibodies for 30 min, washed, and counterstained with 4',6-diamidino-2-phenylindole (Molecular Probes). Labeled cells were visualized using an Olympus Fluoview FV-1000 confocal imaging system on an IX81 platform equipped with a U Plan Fluorite 40×/1.3 NA oil differential interference objective (Olympus).

### Statistical analysis

Data were assessed for symmetry, or skewness, using Pearson’s skewness coefficient. Fold change data were log_2_ transformed to convert the ratio to a difference that better approximates the normal distribution on a log scale. Comparisons between groups were performed using the paired, two-tailed, Student's *t* test. Differences were considered to be statistically significant when *P* < 0.05. * *P* < 0.05; ** *P* < 0.01; *** *P* < 0.001.

## Supporting Information

S1 Fig Purification of HIV. Culture supernatants from HIV_Ba-L_ infected PBMC were clarified through a 0.2 *μ*m filter then concentrated by ultrafiltration through a 300 kDa cutoff filter. Concentrates were then subjected to a 6–18% iodixanol velocity gradient centrifugation and fractions collected. Fraction 1 refers to the top of the gradient as indicated; the protein profiles were analyzed by Ponceau S staining (*top*) or immunoblotting with HIV p24 antibody (*bottom*). M indicates the marker.
(TIF)

S2 Fig Effects of various treatments on LDH leakage. Extracellular release of LDH was measured spectrophotometrically using the Cytotoxicity Detection Kit^PLUS^ during each of the following treatments. (A) Macrophages were exposed to increasing concentrations of cell-free RNase/DNase I treated iodixanol velocity gradient purified HIV for 24 h. *n* = 3. (B) Macrophages were exposed to increasing concentrations of cell-free RNase/DNase I treated iodixanol velocity gradient purified HIV for 24 h in the presence of pepstatin A. *n* = 3. (C) Macrophages were exposed to mock, infectious, AT-2-inactivated, or RNase/DNase I treated AT-2-inactivated iodixanol velocity gradient purified HIV_Ba-L_ for 24 h. *n* = 3. (D) Macrophages transduced with non-specific scrambled shRNA (shNS), or *TLR8* shRNA (sh*TLR8*) were exposed to infectious HIV, AT-2-inactivated HIV, or RNase/DNase I treated AT-2-inactivated HIV or mock infected for 24 h. *n* = 4. (E) Macrophages transduced with shNS or *ATG13* shRNA (sh*ATG13*) were exposed to infectious HIV, 5 *μ*g/mL ssRNA40, 5 *μ*g/mL ssRNA41, or 100 nmol/L sirolimus for 24 h. *n* = 4. (F) Macrophages were pretreated with 100 nmol/L bafilomycin A_1_ then exposed to mock, infectious, or RNase/DNase I treated AT-2-inactivated purified HIV, LyoVec, 5 *μ*g/mL ssRNA41, 5 *μ*g/mL ssRNA40, or 100 nmol/L sirolimus for 24 h. *n* = 3. (G) Macrophages were exposed to HIV_Ba-L_ for 3 h, washed and incubated with fresh media for 10 d. Extracellular release of LDH was measured at days 0, 3, 5, 7, and 10 post-infection. *n* = 6. (H) Macrophages transduced with non-specific cDNA (Control), or *TFEB* cDNA (*TFEB*) then exposed to HIV. Extracellular release of LDH was measured at the indicated times post-exposure. *n* = 4. (I) Macrophages transduced with shNS, *TFEB* shRNA (sh*TFEB*), or *BECN1* shRNA (sh*BECN1*) were infected with HIV_Ba-L_. Extracellular release of LDH was measured at days 0, 3, 5, 7, and 10 post-infection. *n* = 4. (J) Macrophages were exposed to HIV_NL(AD8)_ or HIV_NL(AD8)ΔNef_ for 3 h, washed and incubated with fresh media for 10 d. Extracellular release of LDH was measured at days 0, 3, 5, 7, and 10 post-infection. *n* = 4. Data are reported as mean ± s.e.m.
(TIF)

S3 Fig Inactivation of HIV with AT-2. Macrophages were exposed to HIV or AT-2-treated HIV for 3 h, washed and incubated with fresh media for 10 d. Extracellular release of HIV p24 antigen into the cell supernatant at days 0, 3, 5, 7, and 10 was detected by ELISA. Results are reported as mean ± s.e.m., *n* = 5.
(TIF)

S4 Fig Inhibition of HIV-induced autophagy increases viral replication of Nef deficient HIV. (A) Macrophages were infected with HIV for 3 h, washed and incubated with fresh media for 10 d. Extracellular release of HIV p24 antigen into the cell supernatant at days 0, 3, 5, 7, and 10 was detected by ELISA. Results are reported as mean ± s.e.m., *n* = 5. * *P* < 0.05. (B) Macrophages transduced with non-specific scrambled shRNA (shNS), or TLR8 shRNA (sh*TLR8*), then infected with HIV. ELISA was performed for extracellular release of HIV p24 antigen over 10 d. Results are reported as mean ± s.e.m., *n* = 4.
(TIF)
