# Supplementary figures and images for: Human Immunodeficiency Virus Type 1 Nef Inhibits Autophagy through Transcription Factor EB Sequestration
Source: PLoS Pathog. 2015 Jun 26;11(6):e1005018. doi: 10.1371/journal.ppat.1005018 (PMC4482621; doi:10.1371/journal.ppat.1005018)

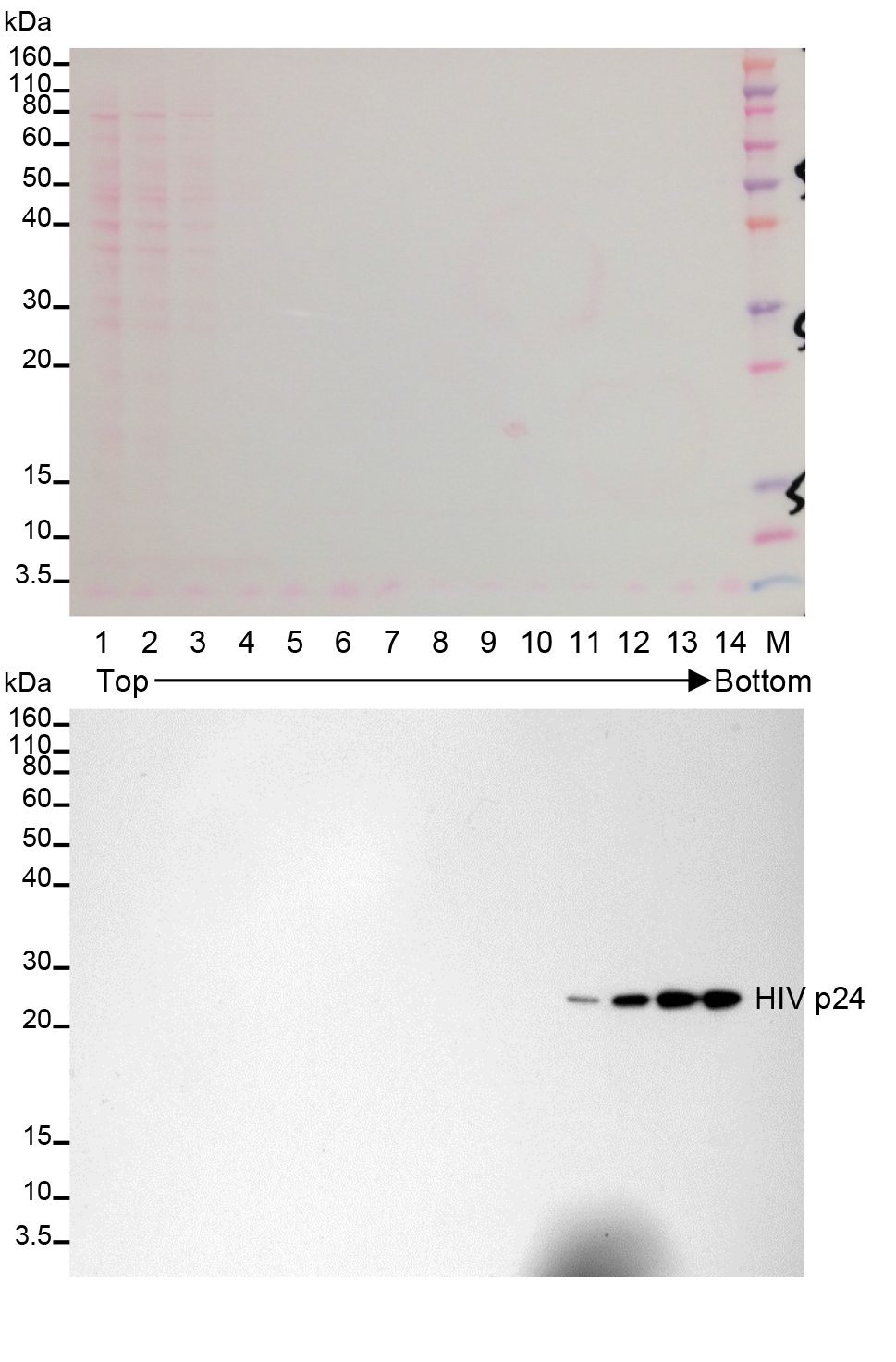

Supplement: S1 Fig — Culture supernatants from HIVBa-L infected PBMC were clarified through a 0.2 μm filter then concentrated by ultrafiltration through a 300 kDa cutoff filter. Concentrates were then subjected to a 6–18% iodixanol velocity gradient centrifugation and fractions collected. Fraction 1 refers to the top of the gradient as indicated; the protein profiles were analyzed by Ponceau S staining (top) or immunoblotting with HIV p24 antibody (bottom). M indicates the marker. (TIF) [file ppat.1005018.s001.tif]

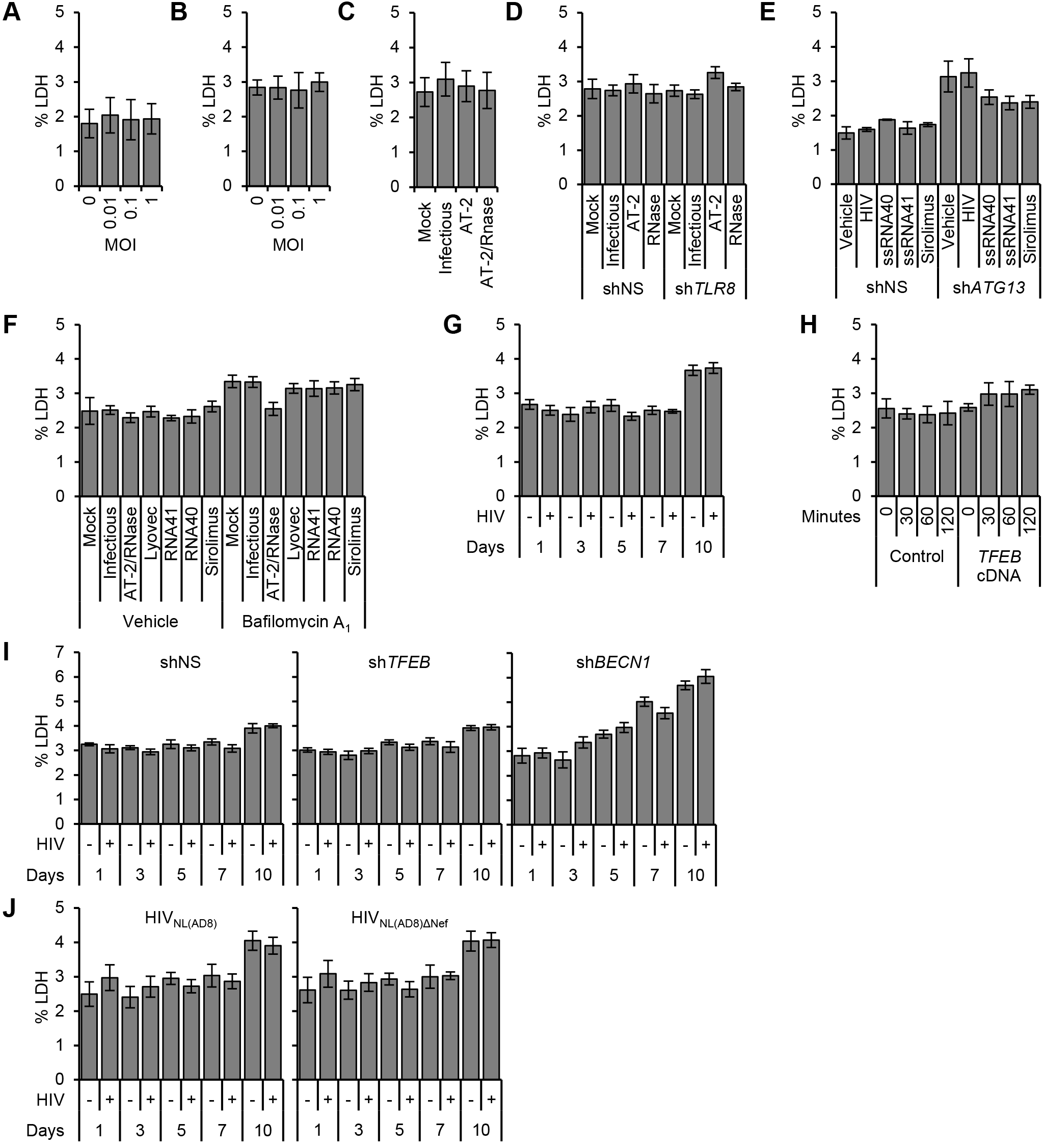

Supplement: S2 Fig — Extracellular release of LDH was measured spectrophotometrically using the Cytotoxicity Detection KitPLUS during each of the following treatments. (A) Macrophages were exposed to increasing concentrations of cell-free RNase/DNase I treated iodixanol velocity gradient purified HIV for 24 h. n = 3. (B) Macrophages were exposed to increasing concentrations of cell-free RNase/DNase I treated iodixanol velocity gradient purified HIV for 24 h in the presence of pepstatin A. n = 3. (C) Macrophages were exposed to mock, infectious, AT-2-inactivated, or RNase/DNase I treated AT-2-inactivated iodixanol velocity gradient purified HIVBa-L for 24 h. n = 3. (D) Macrophages transduced with non-specific scrambled shRNA (shNS), or TLR8 shRNA (shTLR8) were exposed to infectious HIV, AT-2-inactivated HIV, or RNase/DNase I treated AT-2-inactivated HIV or mock infected for 24 h. n = 4. (E) Macrophages transduced with shNS or ATG13 shRNA (shATG13) were exposed to infectious HIV, 5 μg/mL ssRNA40, 5 μg/mL ssRNA41, or 100 nmol/L sirolimus for 24 h. n = 4. (F) Macrophages were pretreated with 100 nmol/L bafilomycin A1 then exposed to mock, infectious, or RNase/DNase I treated AT-2-inactivated purified HIV, LyoVec, 5 μg/mL ssRNA41, 5 μg/mL ssRNA40, or 100 nmol/L sirolimus for 24 h. n = 3. (G) Macrophages were exposed to HIVBa-L for 3 h, washed and incubated with fresh media for 10 d. Extracellular release of LDH was measured at days 0, 3, 5, 7, and 10 post-infection. n = 6. (H) Macrophages transduced with non-specific cDNA (Control), or TFEB cDNA (TFEB) then exposed to HIV. Extracellular release of LDH was measured at the indicated times post-exposure. n = 4. (I) Macrophages transduced with shNS, TFEB shRNA (shTFEB), or BECN1 shRNA (shBECN1) were infected with HIVBa-L. Extracellular release of LDH was measured at days 0, 3, 5, 7, and 10 post-infection. n = 4. (J) Macrophages were exposed to HIVNL(AD8) or HIVNL(AD8)ΔNef for 3 h, washed and incubated with fresh media for 10 d. Extracellular rel [file ppat.1005018.s002.tif]

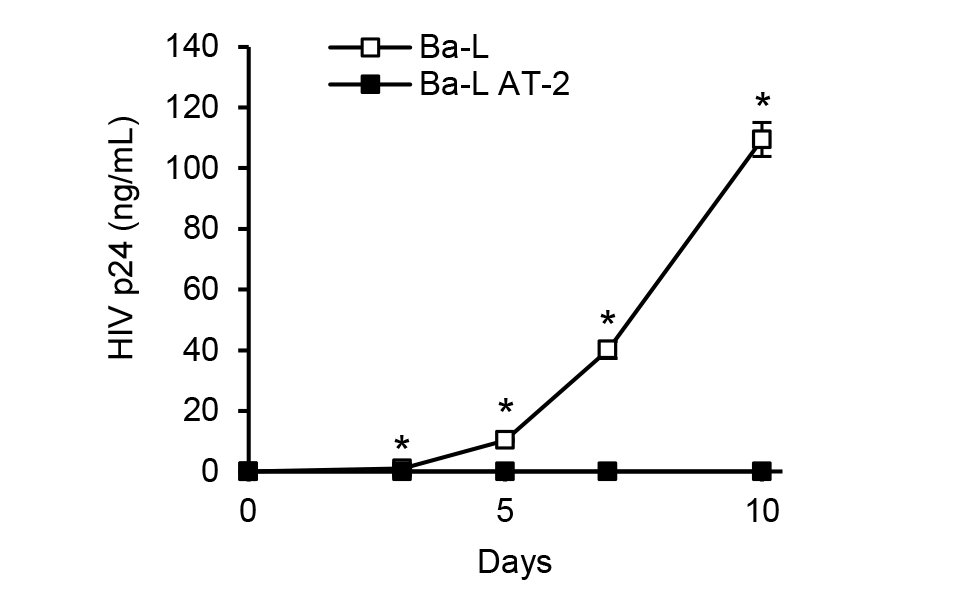

Supplement: S3 Fig — Macrophages were exposed to HIV or AT-2-treated HIV for 3 h, washed and incubated with fresh media for 10 d. Extracellular release of HIV p24 antigen into the cell supernatant at days 0, 3, 5, 7, and 10 was detected by ELISA. Results are reported as mean ± s.e.m., n = 5. (TIF) [file ppat.1005018.s003.tif]

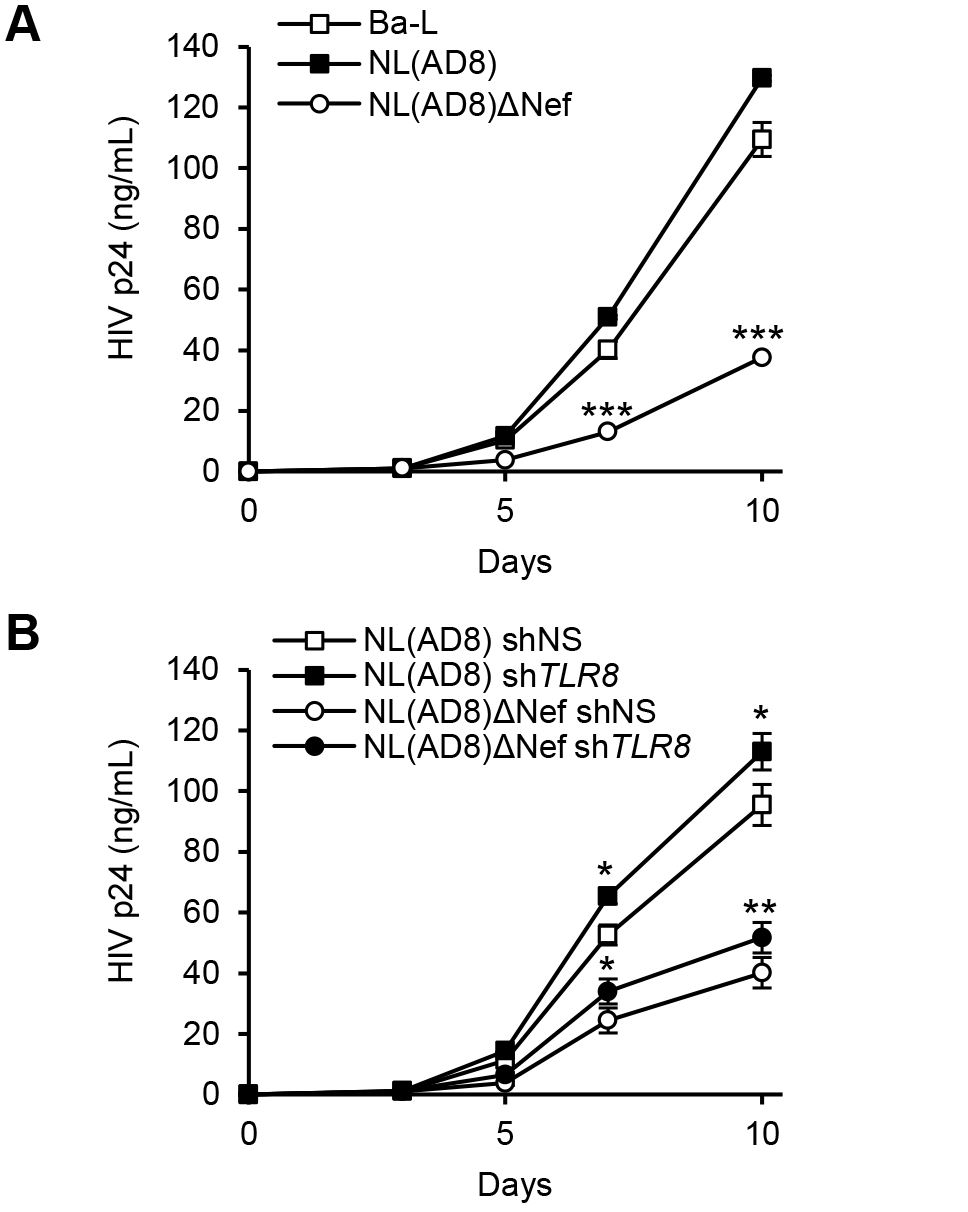

Supplement: S4 Fig — (A) Macrophages were infected with HIV for 3 h, washed and incubated with fresh media for 10 d. Extracellular release of HIV p24 antigen into the cell supernatant at days 0, 3, 5, 7, and 10 was detected by ELISA. Results are reported as mean ± s.e.m., n = 5. * P < 0.05. (B) Macrophages transduced with non-specific scrambled shRNA (shNS), or TLR8 shRNA (shTLR8), then infected with HIV. ELISA was performed for extracellular release of HIV p24 antigen over 10 d. Results are reported as mean ± s.e.m., n = 4. (TIF) [file ppat.1005018.s004.tif]
